# Evaluation of micromotion in multirooted root analogue implants embedded in synthetic bone blocks: an in vitro study

**DOI:** 10.1186/s12903-024-03854-1

**Published:** 2024-01-17

**Authors:** Mostafa Aldesoki, Christoph Bourauel, Tarek M. Elshazly, Erik Schkommodau, Ludger Keilig

**Affiliations:** 1 Dental School, Oral Technology, University Hospital Bonn, Welschnonnenstr.17, 53111 Bonn, Germany; 2 Institute for Medical Engineering and Medical Informatics, University of Applied Sciences and Arts Northwestern Switzerland, Muttenz, Switzerland; 3 Department of Prosthodontics, Dental School, University Hospital Bonn, Bonn, Germany

**Keywords:** Dental implants, Stability, Patient specific, Biomechanics, Motion, 3D printing, Milling, Titanium, Zirconia

## Abstract

**Background:**

While conventional threaded implants (TI) have proven to be effective for replacing missing teeth, they have certain limitations in terms of diameter, length, and emergence profile when compared to customised root analogue implants (RAI). To further investigate the potential benefits of RAIs, the aim of this study was to experimentally evaluate the micromotion of RAIs compared to TIs.

**Methods:**

A 3D model of tooth 47 (mandibular right second molar) was segmented from an existing cone beam computed tomography (CBCT), and a RAI was designed based on this model. Four RAI subgroups were fabricated as follows: 3D-printed titanium (PT), 3D-printed zirconia (PZ), milled titanium (MT), milled zirconia (MZ), each with a sample size of *n* = 5. Additionally, two TI subgroups (B11 and C11) were used as control, each with a sample size of *n* = 5. All samples were embedded in polyurethane foam artificial bone blocks and subjected to load application using a self-developed biomechanical Hexapod Measurement System. Micromotion was quantified by analysing the load/displacement curves.

**Results:**

There were no statistically significant differences in displacement in Z-axis (the loading direction) between the RAI group and the TI group. However, within the RAI subgroups, PZ exhibited significantly higher displacement values compared to the other subgroups (*p* < 0.05). In terms of the overall total displacement, the RAI group showed a statistically significant higher displacement than the TI group, with mean displacement values of 96.5 µm and 55.8 µm for the RAI and TI groups, respectively.

**Conclusions:**

The RAI demonstrated promising biomechanical behaviour, with micromotion values falling within the physiological limits. However, their performance is less predictable due to varying anatomical designs.

## Background

The average survival rate of dental implants after ten years of clinical use is almost 95%, establishing them the best treatment choice for replacing missing and severely decayed teeth [1]. The International Team for Implantology (ITI) consensus conference has classified dental implants based on the insertion protocol as follows: a) immediate implant placement on the day of tooth extraction, b) early implant placement with soft tissue healing typically occurring after 4 to 8 weeks, c) early implant placement with partial bone healing taking place approximately 12 to 16 weeks later, and d) late implant placement after complete bone healing after of least 6 months [2].

Immediate implant placement has many advantages, including shortening the overall treatment time, reducing costs, and decreasing the number of surgical interventions [3]. Additionally, it helps preserving the height and width of the alveolar bone while minimising marginal bone loss following extraction [3–5]. However, the decreased primary stability due to the incongruence between the implant and the alveolus, as well as the difficult implant placement can be a real surgical challenge [6–8].

One of the treatment alternatives to conventional threaded implants (TIs) is the use of fully customised root-analogue implants (RAIs) [9]. The concept of RAI was initially introduced by Hodosh et al. in 1969 as a heat-processed methyl methacrylate implant. However, it was deemed unsuccessful after failure to achieve osseointegration [10]. In 1992, the technique was reintroduced using pure titanium instead of polymer, leading to successful osseointegration [7].

Such RAIs are the product of the combined technologies of computer-aided design/computer-aided manufacturing (CAD/CAM) and cone beam computed tomography (CBCT) [6, 11]. The idea of the RAI is to replace a tooth scheduled for extraction through an immediate implant placement by designing the RAI with similar dimensions to the original root anatomy based on CBCT. Thus, a perfect congruence between the implant and the empty socket could be achieved, unlike TI [12]. The expected benefits include a reduced number of surgeries, simple and straightforward placement, improved primary stability and immediate soft tissue support [13–15].

RAIs are produced using either subtractive or additive manufacturing techniques [16]. Subtractive manufacturing employs a milling process facilitated by computer numerical control (CNC) milling machines. These machines are categorized based on the number of axes they operate on, ranging from 3-axis to 5-axis machines [17, 18]. On the other hand, additive manufacturing involves 3D printing, a method that transforms a digital model into a physical object by depositing materials layer by layer [19, 20].

A notable advancement in additive manufacturing is the use of lithography-based ceramic manufacturing (LCM) for 3D printing advanced ceramics. In this process, a ceramic slurry coated with a photosensitive resin is hardened layer by layer using a light-emitting diode device [21]. Another significant technique in additive manufacturing is selective laser melting (SLM), which involves fusing powdered metal using a high-power fibre laser in an inert environment. The laser precisely melts each layer onto the preceding one, creating a solid object through the accumulation of thousands of micro-welds [22, 23].

The stability of the implant in the alveolar bone is of crucial importance for successful osseointegration [24]. The term micromotion in dental implants refers to the subtle displacement of an implant in relation to the surrounding tissue, which cannot be observed with the naked eye [25]. Studies have suggested that for successful osseointegration, the micromotion between the implant and bone should not exceed a threshold value of 150 µm [26, 27]. Implant stability can be classified into primary and secondary stability. Primary stability is achieved by the mechanical retention of the implant during initial insertion, whereas secondary stability is reached after consecutive bone remodelling processes and complete healing. Consequently, primary stability is considered a mechanical phenomenon, while secondary stability is a biological phenomenon influenced by osseointegration [28]. Many factors influence the primary stability of the implant, such as the quality and quantity of the surrounding bone and the implant geometry; changing the implant-bone contact area by increasing the length or width of the implant could enhance the primary stability [9, 29].

The biomechanical behaviour of dental implants has been extensively investigated in various studies [26, 30, 31]. However, there is a notable gap in research regarding the specific biomechanical behaviour of RAIs. Hence, the aim of this study was to experimentally evaluate the micromotion of multi-rooted titanium and zirconia RAIs by analysing their load/displacement curves. The null hypothesis stated that there would be no statistically significant difference in micromotion among the examined groups or subgroups (*p* > 0.05).

## Methods

### Study design

Two implant designs were used in this study: a custom-designed RAI and a traditional TI as a control. The RAI was designed based on a CBCT scan of a dentate mandible using the following scanning parameters: beam accelerating voltage of 90 kV, X-ray current of 12 mA, voxel dimension of 75 µm, and total scanning time of 15 s. The total number of slices was 668. The CBCT scan was processed using a 3D medical image processing software (Mimics 22; Materialise, Leuven, Belgium) and the right mandibular second molar (tooth 47) was segmented based on histogram analysis. The segmented tooth was subsequently imported into a 3D modelling software (3-matic 15; Materialise, Leuven, Belgium), to finalise the design of the RAI based on the anatomy of the tooth. The coronal portion was designed as an idealised cube with a side length of 5 mm to facilitate further biomechanical investigations.

The RAIs were produced using two methods: milling and 3D printing, with both titanium and zirconia as materials of choice. Milling was performed with 5-axis CNC milling machines, whereas LCM and SLM technologies were used for the 3D printing of zirconia and titanium RAIs, respectively. During 3D printing, the printing supports were placed on the overhanging coronal surface around the idealised cube, and the layer thickness was set to 25 µm. A total of 20 RAIs were fabricated and categorized into four subgroups (*n* = 5) based on the manufacturing method: 3D printed titanium (PT), 3D printed zirconia (PZ), milled titanium (MT), and milled zirconia (MZ).

**Fig. 1:**
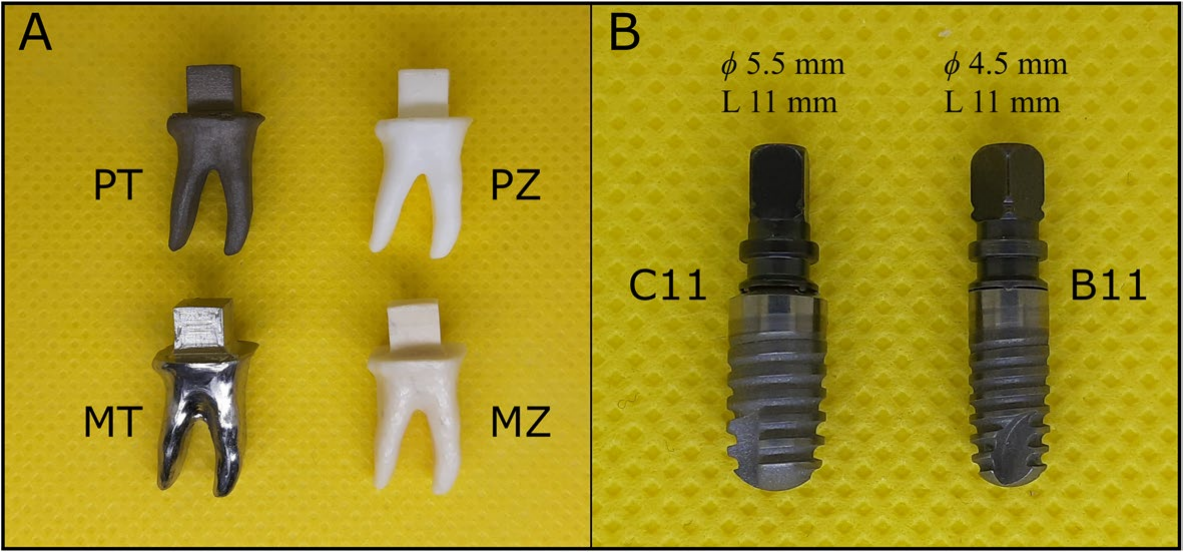
Overview of the examined implant groups. **A** Root analogue implant (RAI) group, including PT, PZ, MT, and MZ subgroups. **B** Threaded Implant (TI) group, comprising C11 and B11 subgroups. Φ, Diameter; L, Length

As a control group, conventional TI were included (Ankylos; Dentsply-Friadent, Mannheim, Germany). The TI group was divided into two subgroups (*n* = 5) based on implant size: B11 subgroup with a diameter of 4.5 mm and length of 11 mm, and C11 subgroup with a diameter of 5.5 mm and length of 11 mm (Fig. 1).

### Biomechanical analysis

**Fig. 2:**
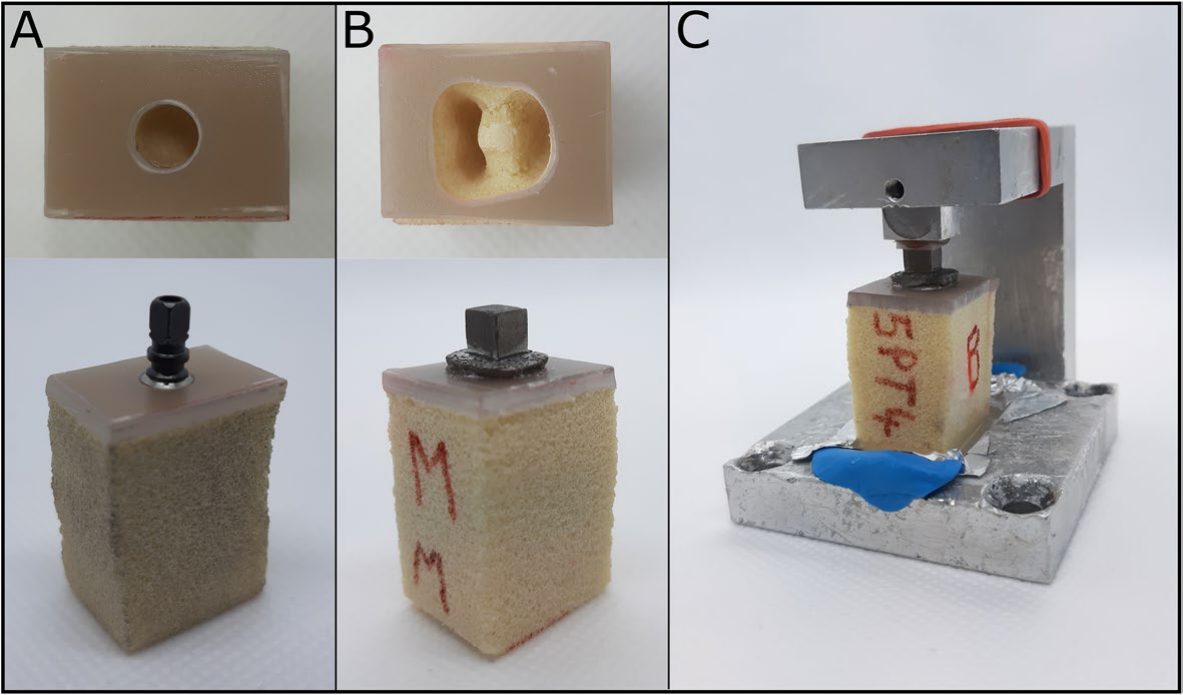
Preparation of specimens for biomechanical testing. **A** Preparation of the osteotomy of the polyurethane foam block to insert the TI. **B** Preparation of the osteotomy of the polyurethane foam block to insert the RAI. **C** Secure fixation of the specimen to the specimen holder using resin. The metal structure on top of the specimen holder holds the aluminium cube in place during preparation, and is removed before measuring the specimen

For the biomechanical testing, the implants were inserted into test blocks made of polyurethane foam artificial bone (Sawbones; Pacific Research Laboratories, Vashon, USA). These test blocks were comprised of two layers: a 2 mm-thick layer of epoxy filled with short glass fibres (type #3401–01, density 1.64 g/cm^3^), which simulated cortical bone, and a 40 mm-thick layer of rigid polyurethane foam (type #1522–01, density 0.16 g/cm^3^), which simulated cancellous bone. The TIs were screwed into Sawbones following the surgical protocol provided by the manufacturer (Fig. 2A). As for the RAIs, a socket-shaped cavity, resembling the negative replica of the root-shaped RAI, was initially drilled in the Sawbones (Fig. 2B). Prior to insertion into the Sawbones, the surface of the RAIs was coated with a thin layer of resin (PalaXpress; Heraeus Kulzer GmbH, Wasserburg, Germany) to secure a tight attachment between the RAI and the Sawbones. Each specimen was then firmly fastened to the base of the specimen holder using PalaXpress resin (Fig. 2C).

**Fig. 3:**
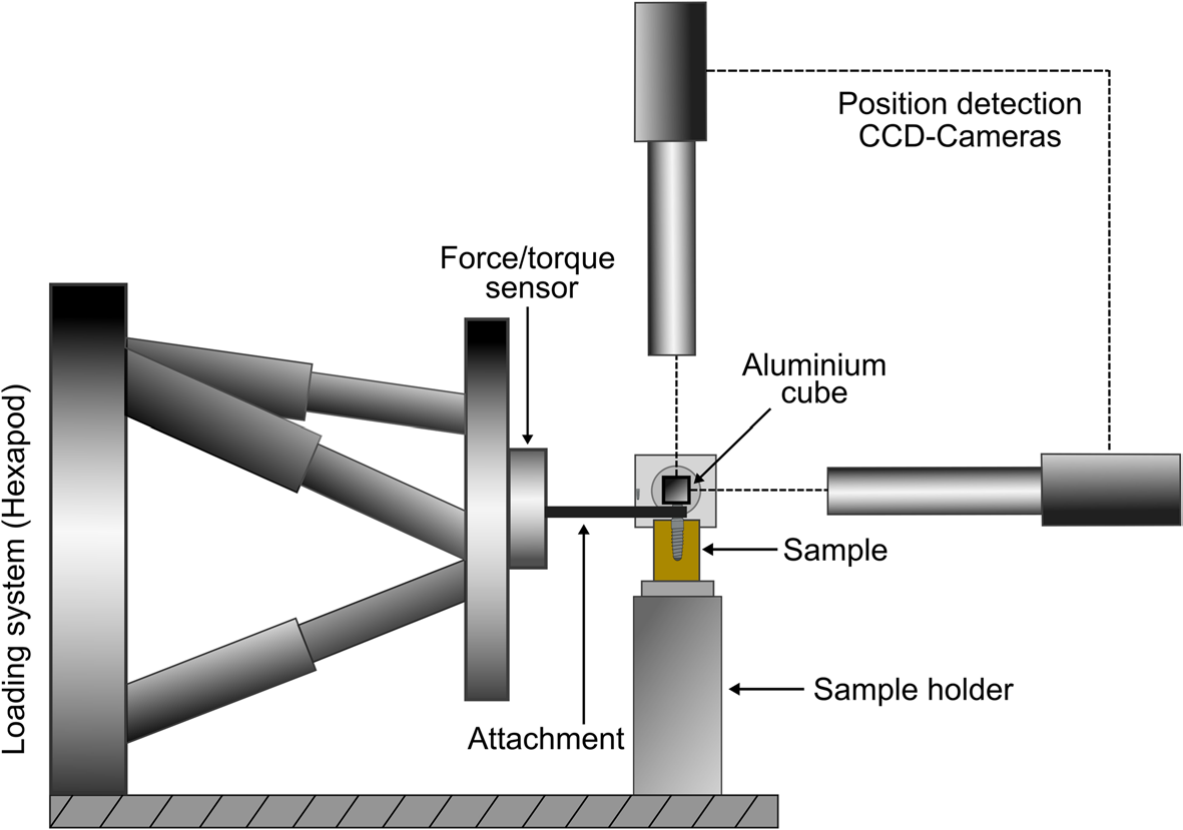
Schematic representation of the Hexapod Measurement System (HexMeS)

The samples were inserted into a custom-developed biomechanical Hexapod Measurement System (HexMeS) [32]. HexMeS is specifically designed to apply various forces on small objects like dental implants. It consists of three main components: a high-precision hexapod robot (PI M-850.50; Physik Instrumente, Karlsruhe, Germany) capable of precise translations and rotations with a resolution of less than 1 µm and 5 µrad. Additionally, the system incorporates a high-precision 3D force/torque sensor (ATI FTSGamma 130/10; SCHUNK GmbH & Co. KG, Lauffen/Neckar, Germany) for force and torque measurements, as well as an optical system for precise position detection, consisting of an aluminium cube with three pinholes, each 2 µm in diameter (Melles-Griot, Bensheim, Germany) illuminated by a laser beam (35 mW, 658 nm; Laser 2000, Wessling, Germany), and three video cameras with macro zoom optics (JAI CV-M1; Stemmer-Imaging, Puchheim, Germany). This setup enables the accurate tracking of micromotions in the specimens under load application by monitoring the pinholes through the video cameras (Fig. 3).

**Fig. 4:**
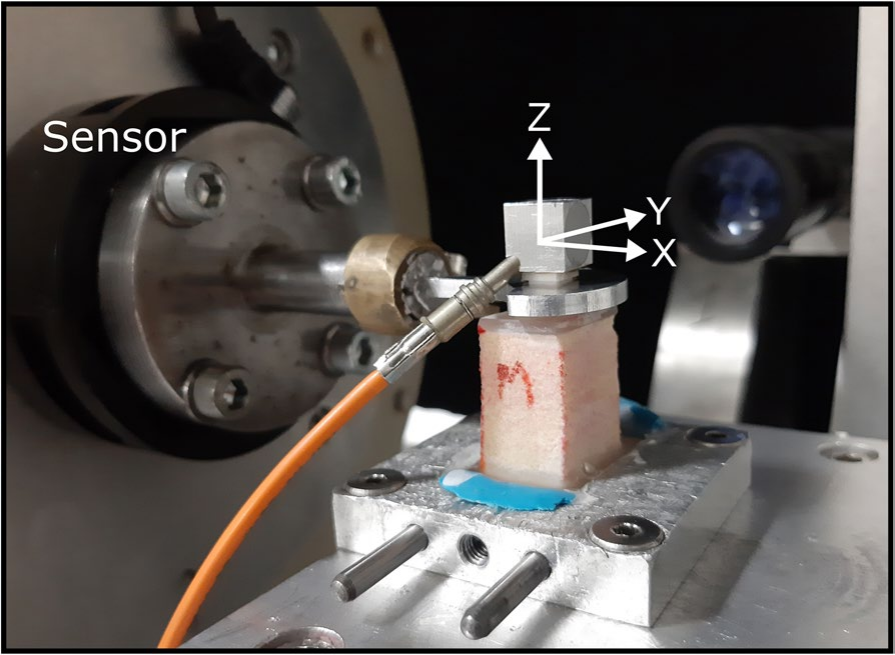
Experimental setup for biomechanical testing. The specimen was securely mounted on the HexMeS system with the implant aligned parallel to the Z axis. An aluminium cube, illuminated by a laser, was attached to the specimen. A loading cycle of 1.5 mm translation in the negative Z direction was applied, followed by a releasing cycle. The resultant force and torque were accurately recorded by the force/torque sensor

The samples were mounted on the HexMeS with the implant aligned parallel to the Z-axis. The laser-illuminated aluminium cube was securely attached to the top of the samples, and a spoon-shaped lever arm was connected to the implant. This configuration allowed any movement of the Hexapod to be transmitted as a force to the implant (Fig. 4).

The samples were indirectly loaded by programming the Hexapod to perform a loading cycle of 1.5 mm translation in the negative Z direction, followed by a release cycle in the positive Z direction until reaching the zero position (150 steps of 0.02 mm each). The applied force and torque were recorded by the force/torque system, and simultaneously the displacement of the implant (translation and rotation) was recorded by tracking the laser-illuminated pinholes through the video cameras. The collected data were exported in CSV format (comma separated values) for further data analysis.

### Statistical analysis

The numerical data are represented as mean values and standard deviations. The normality of the data was assessed using Shapiro–Wilk’s test, while Levene's test was employed to test for homogeneity of variances. The data showed a parametric distribution, homogeneity of variances, and were analysed using nested ANOVA. Estimated marginal means were compared using t-test with p-value adjustment using Tukey’s method. A significance level of *p* ≤ 0.05 was chosen for all statistical tests. The statistical analysis was performed using R statistical analysis software, version 4.1.3 for Windows 1.

## Results

**Fig. 5:**
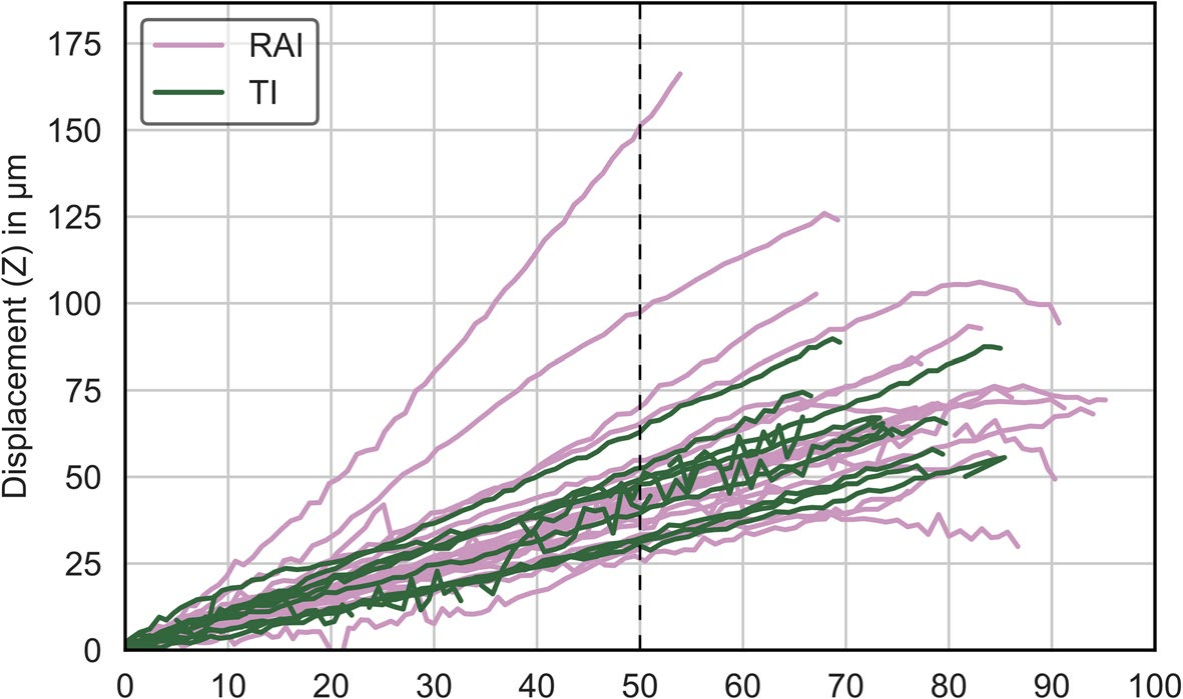
Line chart showing load/displacement curves for all specimens. The dashed line represents the maximum force (50 N) chosen to include all the specimens

The maximum magnitude of forces recorded by the force sensor ranged from 64 to 96 N, whereas the produced maximum displacements ranged from 40 µm to 178 µm. Owing to the different magnitudes of forces, a maximum force of 50 N was chosen for the different specimens to include all specimens (Fig. 5).

The HexMeS allows for the precise measurement of micromovements in each sample, by tracking both the translations and rotations in the three spatial directions. Particular attention will be given to the displacement in the loading direction (Z-axis), as well as the total displacement. The mean values and the standard deviation for all measured displacements in loading direction at a force of 50 N are shown in Table 1.

**Table 1:** Displacement (Z) and total displacement values for both groups and subgroups

Group	Displacement in µm Mean ± SD		Subgroup	Displacement in µm Mean ± SD	
	**(Z)**	**Total**		**(Z)**	**Total**
TI	44 ± 11	56 ± 17	B11	43 ± 8	57 ± 13
			C11	44 ± 14	55 ± 22
RAI	48 ± 18	96 ± 49	PT	39 ± 10	61 ± 7
			PZ	71 ± 22	125 ± 59
			MT	43 ± 0	112 ± 55
			MZ	40 ± 8	88 ± 44

**Fig. 6:**
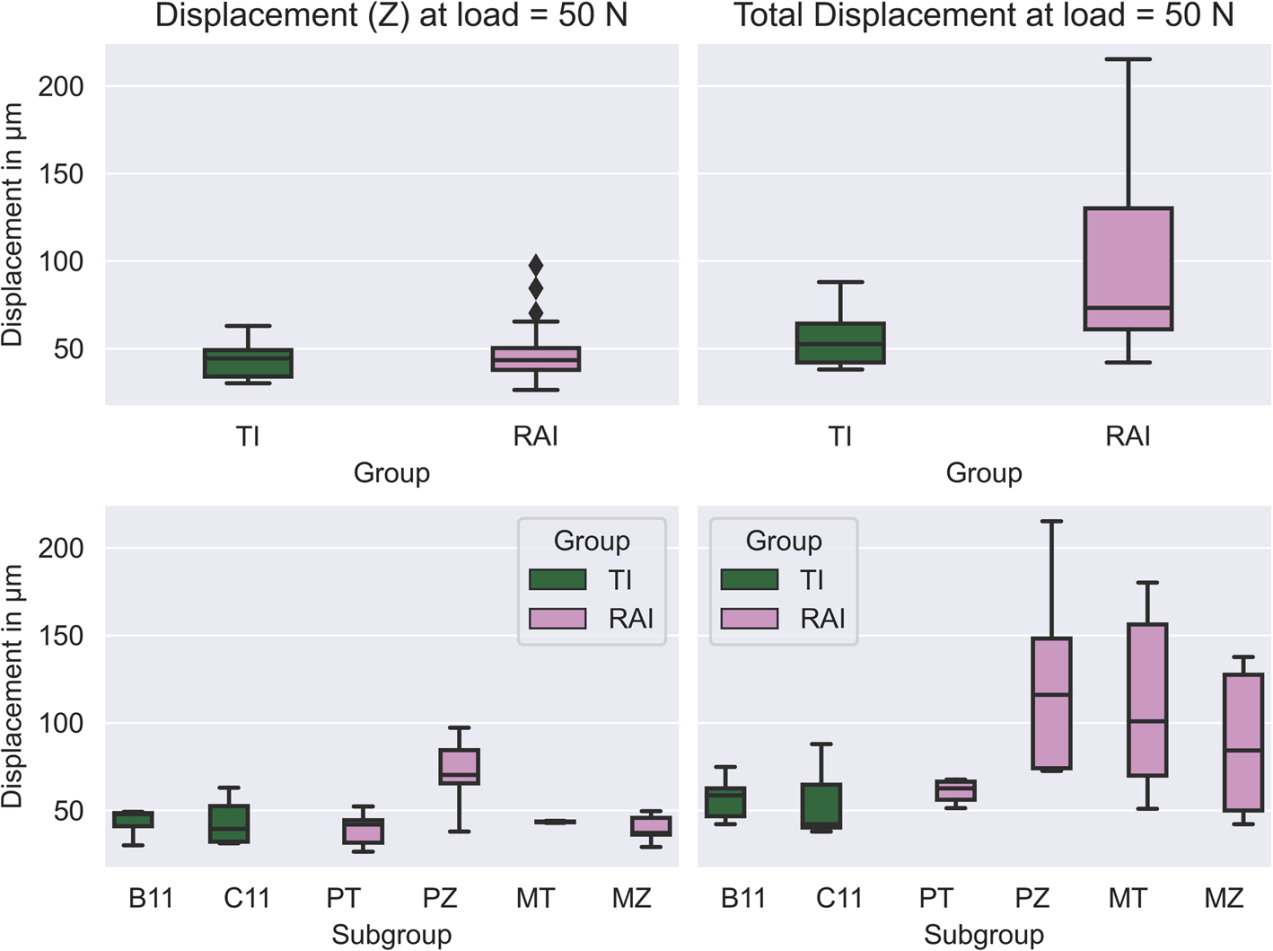
Boxplot diagrams illustrating displacement (Z) and total displacement values across groups and subgroups

The nested ANOVA model for displacement (Z) revealed that there was no statistically significant difference between the two parent groups. The mean displacement for the TI group was 44 µm compared to 48 µm for the RAI group. However, a significant interaction within the nested subgroup variable (*p* = 0.002) was observed. Notably, among the RAI subgroups, PZ exhibited the highest displacement value of 71 µm, while the differences in displacement between the PT, MT, and MZ subgroups were statistically insignificant (Fig. 6 and Tables 1 and 2).

**Table 2:** Nested ANOVA model for displacement (Z) and total displacement

Parameter	Displacement (Z)					Total Displacement				
	**SS**	**DF**	**MS**	***F***	***P***	**SS**	**DF**	**MS**	**F**	***p***
Group	155.61	1	155.61	1.01	0.325	11,046.31	1	11,046.31	7.23	0.013*
Subgroup	3487.78	4	871.94	5.65	0.002*	11,995.26	4	2998.81	1.96	0.133
Error	3703.53	24	154.31			36,664.18	24	1527.67		

*SS* Sum of squares, *DF* Degrees of freedom, *MS *Mean squares, *F* F value, *p* *P* value

^*^Significant (*p* < 0.05)

**Fig. 7:**
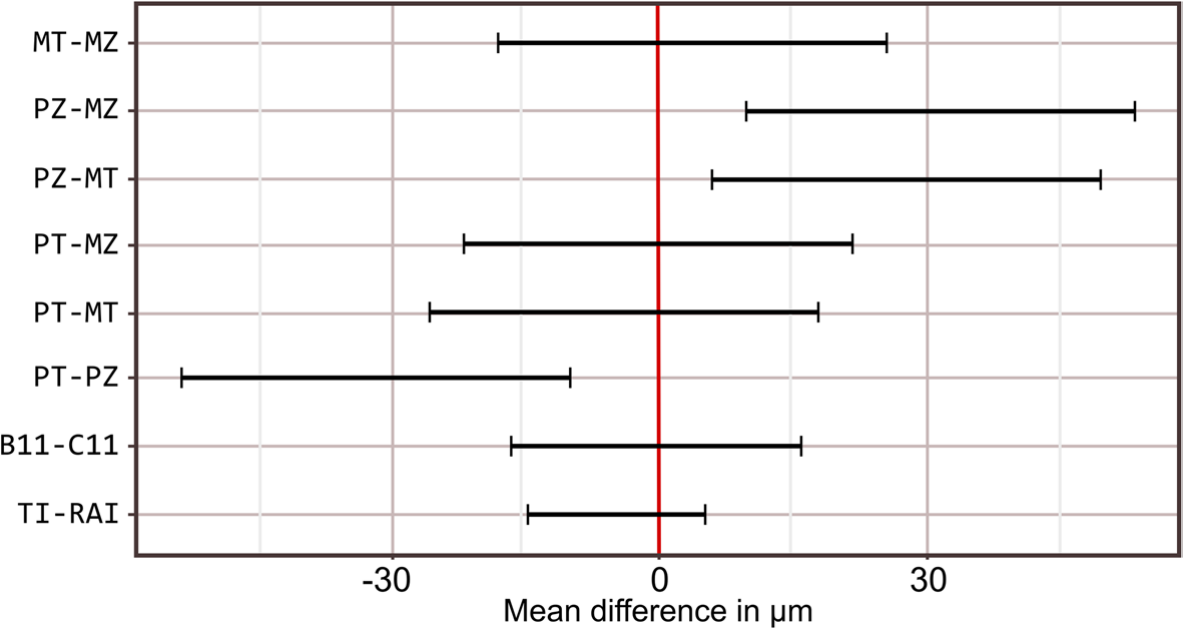
Interval plot showing the variation in estimated marginal means for displacement along the Z-axis

Comparisons of estimated marginal means presented in Table 3 and Fig. 7 indicated that there were no statistically significant differences among the parent groups (*p* = 0.325) or the two subgroups within the TI group (*p* = 0.964). However, within the RAI group, PZ had significantly higher values than the other subgroups (*p* < 0.05).

**Table 3:** Comparison of estimated marginal mean for displacement (Z)

Comparisons	Estimate	95% CI		Statistic	*p* value
		***Lower***	***Upper***		
TI—RAI	-4.83	-14.80	5.10	-1.00	0.325
B11 – C11	-0.35	-16.57	15.86	-0.04	0.964
PT—PZ	-31.75	-53.42	-10.08	-4.04	0.003*
PT—MT	-4.12	-25.8	17.55	-0.53	0.952
PT—MZ	-0.33	-22.01	21.34	-0.04	1.00
PZ—MT	27.63	5.95	49.3	3.52	0.009*
PZ—MZ	31.42	9.75	53.09	4.00	0.003*
MT—MZ	3.79	-17.88	25.46	0.48	0.962

*Significant (*p* < 0.05)

Regarding total displacement, the nested ANOVA model showed that the RAI group had a statistically significantly higher displacement compared to the TI group (*p* = 0.013), with mean displacement values of 96 µm and 56 µm for the RAI and TI groups, respectively. However, the effect of the nested subgroup variable was not statistically significant (*p* = 0.133) (Fig. 6 and Tables 1 and 2).

## Discussion

The objective of this study was to compare the biomechanical behaviour of multi-rooted RAIs and TIs. In vitro load/displacement curves were analysed to assess the micromotion of both implant designs. Based on the study findings, the null hypothesis, which implies no statistically significant difference in micromotion among the examined groups or subgroups, was partially rejected.

One of the determining key factors for successful implant placement is implant stability, whether it is assessed immediately after implant placement or after osseointegration. The stability of dental implants has been evaluated in literature by various methods including Periotest® and resonance frequency analysis [29]. In the present in vitro study, we have used micromotion as an indicator of implant stability by comparing the produced displacements of TI and RAI under specific loading conditions [31, 33].

Our objective was to replicate the clinical process of placing RAI by utilizing CBCT data acquisition and CAD software to design the RAI according to the natural root shape of tooth 47 [34]. We have specifically chosen a multirooted tooth to explore the intricate mechanical characteristics of such teeth, which have not been thoroughly examined in prior research. The two most common materials used in the manufacturing of RAIs are titanium alloy and zirconia [16, 35]. Owing to the biocompatibility and the remarkable mechanical and physical properties of titanium, it has been widely used for dental implants [36]. Nonetheless, the increasing emphasis on aesthetics has led to the emergence of zirconia as a viable alternative [37]. Zirconia exhibits high biocompatibility, superior flexural strength, reduced bacterial affinity, and the advantage of adjustable white colour [36]. In our study, both titanium alloy and zirconia were selected as materials for the RAIs, using both additive and subtractive manufacturing methods.

Sawbones artificial bone blocks were used instead of cadaver bone to take advantage of their uniform and standardised physical properties. This reduced variability and eliminated the special handling requirements associated with cadaver bone. PalaXpress resin was chosen to fix the RAIs in the Sawbones, owing to its appropriate working time, stability, and radiopacity, as previously reported in the literature [31]. In contrast, the TIs were firmly inserted into the drilled Sawbones without requiring any resin application.

The results of this study revealed that there was no statistically significant difference in displacements along the loading direction (Z-axis) between the RAIs and the TIs, suggesting comparable stability between the two implant types. These findings are consistent with a study by Gattinger et al. [38], where they compared by finite element analysis the micromotion of RAI and standard implant and reported that the RAI was as good as the standard threaded implant in terms of micromotion. A similar conclusion was reached out by Chen et al. [39] who studied the biomechanical performance of RAI for both the immediate and the delayed loading protocols. They observed increased micromotion in the RAI during immediate loading, but reduced micromotion during the osseointegrated phase with bonded contact simulation, indicating reliable long-term stability.

Based on the findings of a previous study conducted by Aldesoki et al. [34], it was observed that the manufacturing method had a slight impact on the dimensions of the produced RAIs. Taking this into consideration, our study incorporated four RAI subgroups that comprised different combinations of manufacturing techniques and materials. The analysis of estimated marginal means revealed a statistically significant higher displacement in the PZ subgroup compared to the other RAI subgroups (*p* > 0.5). This observation aligns with the aforementioned study, which reported noticeable warpage at the apical part of the RAI during the manufacturing process specifically in the PZ group [34]. Such warpage may contribute to increased susceptibility of the RAI to displacement or movement under loading conditions.

Regarding total displacement, the RAI group exhibited a statistically significant increase in displacement compared to the TI group (*p* < 0.05). Specifically, the RAI demonstrated increased micromotion along the X and Y axes, while the TI group primarily experienced micromotion along the loading Z axis. This eccentric micromotion behaviour of the RAI can be attributed to its anatomical shape, characterised by asymmetric mesial and distal roots in terms of form and length. Additionally, the experimental loading conditions of the HexMeS setup, where the specimens are indirectly loaded through the spoon-shaped attachment, indirectly contribute to this behaviour. From a biomechanical perspective, the spoon-shaped attachment acts as a lever arm, generating torque on the implant and resulting in rotation around the Y axis, thereby increasing displacement along the X axis.

Noteworthy, the mean micromotion values observed in the RAI were approximately 48 µm for displacement along the Z-axis and approximately 96 µm for total displacement. These values remain below the maximum threshold value of micromotion crucial for successful osseointegration, which is estimated to be around 150 µm [26, 27]. These findings indicate that the range of micromotion exhibited by the RAI is unlikely to impede the osseointegration process.

We presume that this study has effectively investigated the stability of RAI by closely adhering to the clinical workflow and utilizing the latest technologies in RAI preparation. Nevertheless, certain limitations should be considered. Firstly, this is an in vitro study, thus the histological examination of osseointegration was not feasible. Secondly, the fixation of RAI in the artificial bone involved the use of a thin resin layer, which was a crucial step owing to the anatomical shape of RAI and the non-drilling surgical protocol. Finally, the loading of the HexMeS setup could not be done directly on the specimens, and the spoon-shaped load applicator might have introduced an additional torque.

In view of the results of the present study, RAIs showed biomechanical behaviour in terms of stability and micromotion comparable to that of TIs. Moreover, based on previous studies [14, 34], RAIs fabricated by milling or 3D printing showed promising results in terms of dimensional accuracy. Collectively, these findings propose that RAI could serve as a feasible alternative to TI, particularly in immediate implant cases, provided a well-prepared preoperative treatment plan and access to a capable CBCT device. However, it's crucial to note that the tooth to be replaced should lack sharp undercuts that might impede the insertion of the RAI or compromise its proper fit. Nevertheless, further clinical trials and studies are necessary to validate its clinical application.

## Conclusions

After acknowledging the limitations of this study, we drew the following conclusions:

1. The RAI exhibited promising biomechanical behaviour, as indicated by micromotion values within physiological limits.
2. The stability of the RAI could be influenced by the manufacturing technique.
3. Compared to the TI, the biomechanical behaviour of the RAI is less predictable due to its irregular anatomical design.
4. Precise definition of the implant geometry is essential to ensure a precise fit and a seamless insertion.

## Data Availability

The datasets used and/or analysed during the current study available from the corresponding author on reasonable request.

## Abbreviations

TI: Threaded implant
RAI: Root analogue implant
CBCT: Cone beam computed tomography
ITI: International team for implantology
CAD/CAM: Computer-aided design/Computer-aided manufacturing
CNC: Computer numerical control
LCM: Lithography-based ceramic manufacturing
SLM: Selective laser melting
HexMeS: Hexapod measurement system
CSV: Comma separated values

## Footnotes

1 R Core Team (2022). R: A language and environment for statistical computing. R Foundation for Statistical Computing, Vienna, Austria. URL https://www.R-project.org/
